# WNT signaling suppresses oligodendrogenesis via Ngn2-dependent direct inhibition of Olig2 expression

**DOI:** 10.1186/s13041-020-00696-0

**Published:** 2020-11-13

**Authors:** Min Jiang, Dan Yu, Binghua Xie, Hao Huang, Wenwen Lu, Mengsheng Qiu, Zhong-Min Dai

**Affiliations:** Institute of Life Sciences, Key Laboratory of Organ Development and Regeneration of Zhejiang Province, College of Life Sciences, Hangzhou Normal University, Hangzhou, 310029 People’s Republic of China

**Keywords:** WNT, β-catenin, Oligodendrocyte, *Ngn2*, *Olig2*

## Abstract

*Olig2* transcription factor is essential for the maintenance of neural progenitor cells (NPCs) in the pMN domain and their sequential specification into motor neurons (MNs) and oligodendrocyte precursor cells (OPCs). The expression of *Olig2* rapidly declines in newly generated MNs. However, *Olig2* expression persists in later-born OPCs and antagonizes the expression of MN-related genes. The mechanism underlying the differential expression of *Olig2* in MNs and oligodendrocytes remains unknown. Here, we report that activation of WNT/β-catenin signaling in pMN lineage cells abolished *Olig2* expression coupled with a dramatic increase of *Ngn2* expression. Luciferase reporter assay showed that *Ngn2* inhibited *Olig2* promoter activity. Overexpression of Ngn2-EnR transcription repressor blocked the expression of *Olig2 *in ovo. Our results suggest that down-regulation of WNT-*Ngn2* signaling contributes to oligodendrogenesis from the pMN domain and the persistent *Olig2* expression in OPCs.

*Olig2* is the key transcription factor that not only maintains the neural progenitor cells (NPCs) of pMN domain, but also regulates the sequential specification of NPCs into motor neurons (MNs) and OPCs [1–5]. Since persistent expression of *Olig2* is inhibitory to post-mitotic MN genes [6], the expression of *Olig2* rapidly declines in newly generated MNs, but remains high in later-born cells of oligodendrocyte lineage [2–6]. The mechanism of down-regulation of *Olig2* expression in MNs remains elusive. WNT signaling is known to regulate the balance between the proliferation and differentiation of NPCs during neurogenesis [7]. It is interesting that endogenous WNT/β-catenin signaling is activated in newly generated MNs [8]. Activation of WNT/β-catenin signaling has been reported to inhibit the specification of OPCs and astrocytes from NPCs during early stages of gliogenesis [9–11]. However, the mechanism underlying the inhibition of OPC specification from pMN NPCs by WNT/β-catenin signaling remains to be determined.

Here, we utilized the *Olig1*^Cre/+^;*Ctnnb1*^ΔEx3/+^ transgenic mice to activate WNT signaling in the pMN domain. At embryonic day 12.5 (E12.5) when oligodendrogenesis commences, expression of *Olig1* and *Olig2* remains high in the pMN neural progenitor cells from which *Pdgfra*+ OPCs arise (Fig. 1a). Strikingly, in *Olig1*^Cre/+^;*Ctnnb1*^ΔEx3/+^ transgenic mice, activation of WNT signaling totally abolished the expression of *Olig1*, *Olig2* and *Pdgfra* (Fig. 1a), indicating a complete inhibition of oligodendrogenesis. By contrast, the number of ISL1-positive MNs was only decreased slightly in *Olig1*^Cre/+^;*Ctnnb1*^ΔEx3/+^ mice (Additional file 1: Fig. S1), consistent with the previous finding that *Olig1* is intermittently expressed in pMN NPCs and only weakly expressed during neurogenesis stage [12]. Although *Olig1*^Cre^ was also transcribed in P3 domain at early stages [13, 14], expression of P3 domain marker NKX2-2 was not suppressed in *Olig1*^Cre/+^;*Ctnnb1*^ΔEx3/+^ mice (Additional file 1: Fig. S1). However, the number of *Ngn2*-positive cells was dramatically increased within the ventral ventricular region in *Olig1*^Cre/+^;*Ctnnb1*^ΔEx3/+^ mice (Fig. 1a), demonstrating that WNT activation promotes *Ngn2* expression. In support of this notion, overexpression of *Ctnnb1*^ΔEx3^ in embryonic chicken spinal cord also caused an increase of *Ngn2* expression, coupled with a reduced expression of *Olig2* (Fig. 1d). At E18.5, although a few dorsally-derived [15–17] OPCs were generated from *Olig1*^Cre/+^;*Ctnnb1*^ΔEx3/+^ mice, *Plp1*-positive mature oligodendrocytes were still undetectable (Additional file 1: Fig. S2) since dorsal OPCs differentiate only after birth [14]. Together, these results strongly suggest that *Ngn2* is the candidate gene that mediates the suppression of oligodendrogenesis from pMN NPCs by WNT signaling.

**Fig. 1:**
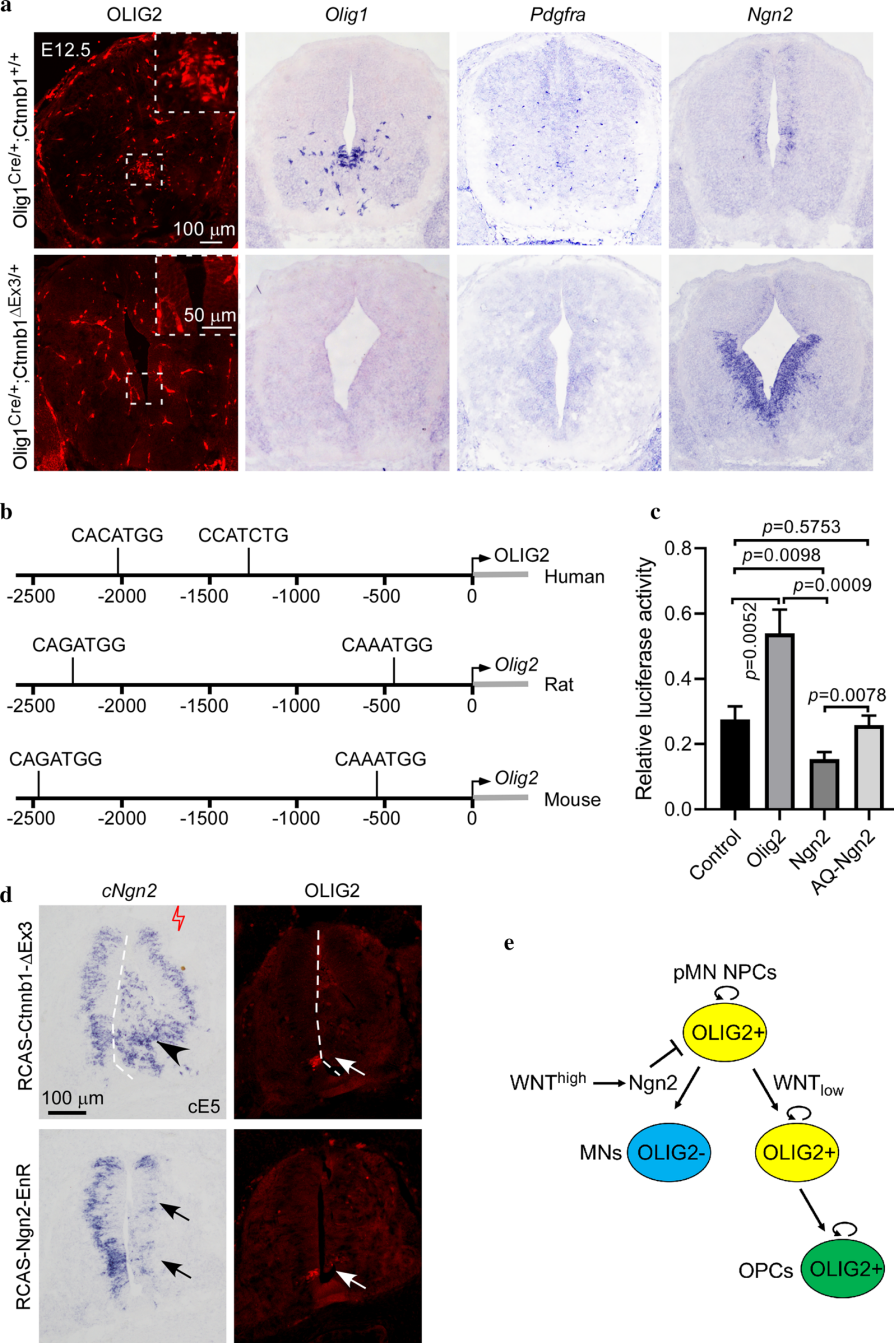
WNT signaling inhibit *Olig2* expression through upregulation of *Ngn2* expression. **a** Transverse sections of spinal cord at E12.5 from control and WNT signaling activated (*Olig1*^Cre/+^;*Ctnnb1*^ΔEx3/+^) mice were subjected to IF with anti-OLIG2 antibody or ISH with *Olig1*, *Pdgfrα* and *Ngn2* riboprobes. The cells positive for OLIG2, *Olig1* and *Pdgfrα* are absent in the spinal cord from *Olig1*^Cre/+^;*Ctnnb1*^ΔEx3/+^ mice, whereas *Ngn2* is upregulated. Inset highlights the expression of OLIG2 in pMN domain, note that vascular development was abnormal in the spinal cord of *Olig1*^Cre/+^;*Ctnnb1*^ΔEx3/+^ mice. **b** There are putative *Ngn2* binding sequences in the promoter regions of *Ngn2* from human, rat and mouse. **c** Luciferase report assay revealed that *Ngn2* but not its DNA binding deficient mutant AQ-*Ngn2* inhibit the promoter activity of mouse *Olig2*. *p < 0.05, t-test. **d** Over-expression of *Ngn2*-EnR mimics the phenotype caused by over-expression of *Ctnnb1*-ΔEx3. Both expression of *Ctnnb1*-ΔEx3 and *Ngn2*-EnR suppressed the expression of OLIG2 *in ovo*. Arrowhead indicates induced expression of chick *Ngn2* (cNgn2). Arrows represent reduced expression of endogenous genes. **e** OLIG2 maintains proliferation of pMN domain neural progenitor cells. High level of WNT signaling upregulates *Ngn2* expression, NGN2 in turn coordinate with OLIG2 to promote motor neurons specification and suppress *Olig2* expression in newly generated motor neurons. OPCs were specified OLIG2+ cells when WNT signaling is declined at the gliogenesis stage

In line with this concept, two NGN2 recognition sequences are identified in the upstream promoter of the *Olig2* gene in human, rat and mouse (Fig. 1b). Luciferase reporter assay revealed that *Ngn2* but not its DNA binding defective mutant AQ-*Ngn2* can inhibit the promoter activity of mouse *Olig2* (Fig. 1c), demonstrating that *Ngn2* can bind to the promoter of *Olig2* and repress its expression. To confirm that *Ngn2* mediates WNT inhibition of oligodendrogenesis, we overexpressed *Ngn2* in embryonic chicken spinal cord by in ovo electroporation and found a significant decrease of *Olig2* and *Pdgfra* expression in the electroporated side at cE7 (Additional file 1: Fig. S3). Since *Ngn2* can function either as a transcriptional activator or a repressor, we next investigated whether the inhibition of *Olig2* expression is mediated by the transcriptional repressor activity of *Ngn2*. RCAS-Ngn2-EnR (DNA binding domain of NGN2 fused with EnR transcription repressor) was employed as a repressor-only NGN2 chimeric protein. It was found that overexpression of this chimeric repressor caused a significant reduction of *Olig2* expression (Fig. 1d), mimicking the effect of full-length *Ngn2* protein. This finding demonstrated that *Ngn2* inhibits *Olig2* expression by its transcriptional repressor activity.

In conclusion, our results suggest that WNT signaling up-regulates the expression of *Ngn2*, and *Ngn2* in turn inhibits *Olig2* expression and oligodendrogenesis during MN specification (Fig. 1e).

## Supplementary information

**Additional file 1:** Supplementary materials and results.

## Data Availability

All date generated during this study are included in this article.
