# Supplementary material for: WNT signaling suppresses oligodendrogenesis via Ngn2-dependent direct inhibition of Olig2 expression
Source: Mol Brain. 2020 Nov 13;13:155. doi: 10.1186/s13041-020-00696-0 (PMC7666497; doi:10.1186/s13041-020-00696-0)
Supplement: Supplementary file 1 — Additional file 1: Supplementary materials and results. [file 13041_2020_696_MOESM1_ESM.docx]

**Materials and methods**

**Animals.** The use of animals was approved by the Committee on Laboratory Animals, Hangzhou Normal University. *Olig1^Cre^* with the *Neo* cassette has been previously described ([1](#_ENREF_1)). The mouse line β-catenin^loxP(Exon3)^ ([2](#_ENREF_2)) were obtained from the Jackson Laboratory, Bar Harbor, ME. Mice with conditional WNT/β-catenin activation (Ctnnb1^ΔEx3/+^) resulting from stabilized β-catenin were obtained by crossing *Olig1^Cre^* with β-catenin^flox(Exon3)^ mice. The Olig1^Cre/+^ (with Neo);β-catenin^ΔExon3/+^ mice died perinatally. Phenotypes are more severe than those observed in *Olig1^Cre^*^/+^ (with the *Neo* cassette deleted);β-catenin^ΔExon3/+^ ([3](#_ENREF_3)), which is possibly due to the earlier expression of *Olig1^Cre^* with *Neo* than *Olig1^Cre^* without *Neo* ([4](#_ENREF_4), [5](#_ENREF_5)).

**In situ hybridization and immunofluorescence**

Samples were fixed overnight in 4% paraformaldehyde/PBS at 4^o^C followed by 20% sucrose infusion, embedded in Tissue-Tek® O.C.T™ Compound (Sakura Finetek), and stored at -80^o^C. Samples were cryosectioned at 18 μm for ISH. DIG-labeled RNA probes were transcribed by T7, T3, or SP6 RNA polymerase using DIG RNA Labeling Mix (Roche Diagnostics). Standard ISH was performed according to the manufacturer’s instructions.

Tissue sections at 14 μm were used for immunofluorescent staining. Sections were dried at room temperature for 20 min., and then incubated in PBS for 5 min blocked in blocking buffer (3% BSA and 0.1% Triton X100 in TBS) for 30 min at room temperature before primary antibodies were applied overnight at 4^o^C. After washing three times in TBS, sections were incubated with the fluorescent secondary antibody at 1:3,000 at room temperature for 1 hr, washed three times in TBS, and then proceeded to mounting. The following primary antibodies and their corresponding secondary antibodies were used: anti-OLIG2 (Millpore, MABN50, 1:500), goat-anti-mouse IgG2a (Invitrogen, A-21135); anti-ISL1 (Developmental Studies Hybridoma Bank, 40.2D6, 1:200), goat-anti-mouse IgG1 (Invitrogen, A-21125); anti-Nkx2.2 (Developmental Studies Hybridoma Bank, 74.5A5, 1:100), goat-anti-mouse IgG2b (Invitrogen, A-21141); anti-β-catenin (Abcam, ab16051, 1:200), goat-anti-Rabbit IgG(H+L) (Invitrogen, A-11012).

**Luciferase report assay**

For luciferase activity assay, the 2.6 kb upstream sequence from mouse Olig2 transcription start site was amplified and inserted into the NheI and XhoI sites of pGL3-enhancer vector (pGLe, Promega) by T4 DNA polymerase ([6](#_ENREF_6), [7](#_ENREF_7)). Flag-tagged Olig2, Ngn2 and AQ-Ngn2 were cloned into pCDH-CMV-MCS-EF1-copGFP (pCDH) as previous ([8](#_ENREF_8)). Each 1 μg of pGL3-enhancer was mixed with 0.1 μg of pRL-TK (Promega), and 0.1 μg of either pCDH, pCDH-Olig2, pCDH-Ngn2 or pCDH-AQ-Ngn2 for transfection into HEK293T cells. Luciferase activities were measured using Dual-Luciferase® Reporter Assay System (Promega).

***In ovo* electroporation and tissue section**

We used retrovirus vector RCASBP containing β-catenin-ΔExon3, Ngn2 or Ngn2-EnR (Ngn2 DNA binding domain fused with EnR domain) for electroporation. Briefly, fertilized eggs were incubated at 38.5°C, with a humidity of 60-70% until chicken embryonic day 2 (cE2), after which 3‐4 mL of albumin was removed without disrupting the yolk. The plasmid was adjusted to 2 µg/µl of target gene containing vector mixed with 0.8 µg/µl of pCAGGS-EGFP. Egg shell was cut carefully to construct a 1‐2 cm diameter window. The plasmid mixture was injected into the neural tube. Electrodes were then immediately placed in parallel on each side of the spinal cord. A total of 25‐volt pulses, lasting 50 ms and separated by a 50 ms pause, were emitted. Following electroporation, the electrodes were carefully removed, and the eggs were sealed with tape and allowed to develop further in the incubator until they reach the desired stage (cE5 or cE7).

**References**

1. Lu QR, Sun T, Zhu Z, Ma N, Garcia M, Stiles CD, et al. Common developmental requirement for Olig function indicates a motor neuron/oligodendrocyte connection. Cell. 2002;109(1):75-86.

2. Harada N, Tamai Y, Ishikawa T, Sauer B, Takaku K, Oshima M, et al. Intestinal polyposis in mice with a dominant stable mutation of the beta-catenin gene. The EMBO journal. 1999;18(21):5931-42.

3. Dai ZM, Sun S, Wang C, Huang H, Hu X, Zhang Z, et al. Stage-specific regulation of oligodendrocyte development by Wnt/beta-catenin signaling. The Journal of neuroscience : the official journal of the Society for Neuroscience. 2014;34(25):8467-73.

4. Paes de Faria J, Kessaris N, Andrew P, Richardson WD, Li H. New Olig1 null mice confirm a non-essential role for Olig1 in oligodendrocyte development. BMC neuroscience. 2014;15:12.

5. Xin M, Yue T, Ma Z, Wu FF, Gow A, Lu QR. Myelinogenesis and axonal recognition by oligodendrocytes in brain are uncoupled in Olig1-null mice. The Journal of neuroscience : the official journal of the Society for Neuroscience. 2005;25(6):1354-65.

6. Sun S, Guo W, Yang JS, Qiu M, Zhu XJ, Dai ZM. TT(N)mGCCTC inhibits archaeal family B DNA polymerases. Scientific reports. 2018;8(1):1990.

7. Sun S, Huang H, Qi YB, Qiu M, Dai ZM. Complementary annealing mediated by exonuclease: a method for seamless cloning and conditioning site-directed mutagenesis. Biotechnology, biotechnological equipment. 2015;29(1):105-10.

8. Sun S, Zhu XJ, Huang H, Guo W, Tang T, Xie B, et al. WNT signaling represses astrogliogenesis via Ngn2-dependent direct suppression of astrocyte gene expression. Glia. 2019;67(7):1333-43.


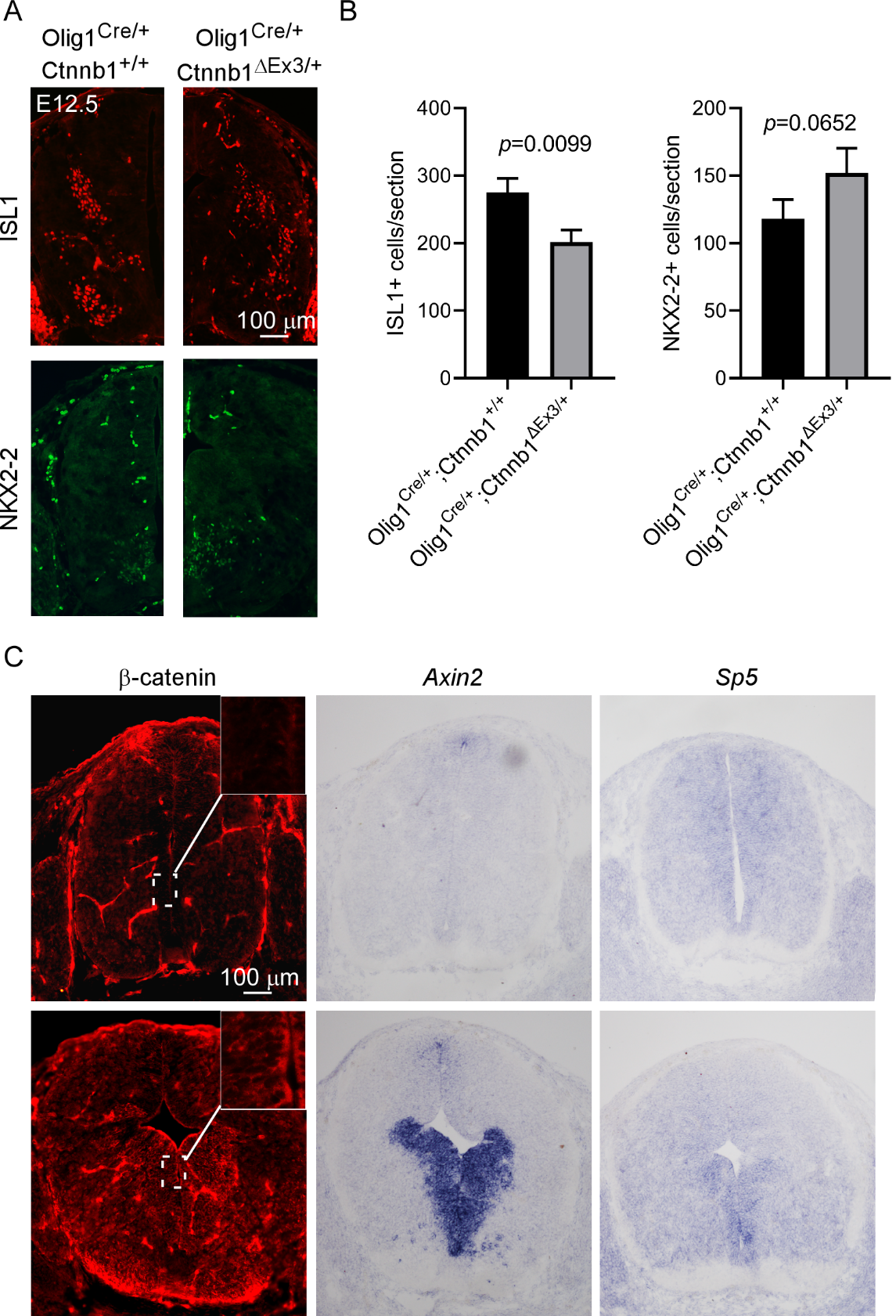


**Figure S1.** (A-B) Activation of β-catenin by *Olig1*^Cre/+^ resulted in a decreased number of ISL1+ motor neurons generated from pMN domain NPCs, but NKX2-2+ interneurons was not reduced. **: *p*<0.01; ns: not significant. (C) Immunofluorescence of β-catenin and in situ hybridization of canonical WNT signal targets Axin2 and Sp5 revealed activation of WNT signaling in the *Olig*^Cre/+^;β-catenin^ΔExon3/+^ mice.


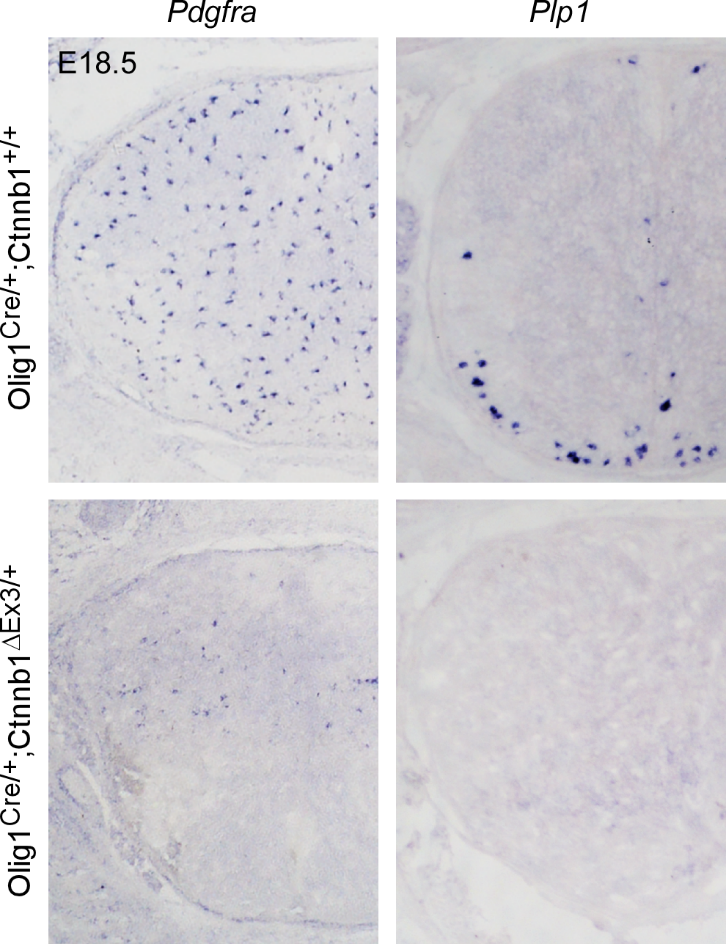


**Figure S2.** Only a small number of dorsally-derived OPCs were observed in *Olig*^Cre/+^;β-catenin^ΔExon3/+^ mice at E18.5. *In situ* hybridization of OPC marker *Pdgfra* and mature oligodendrocyte marker *Plp1*.


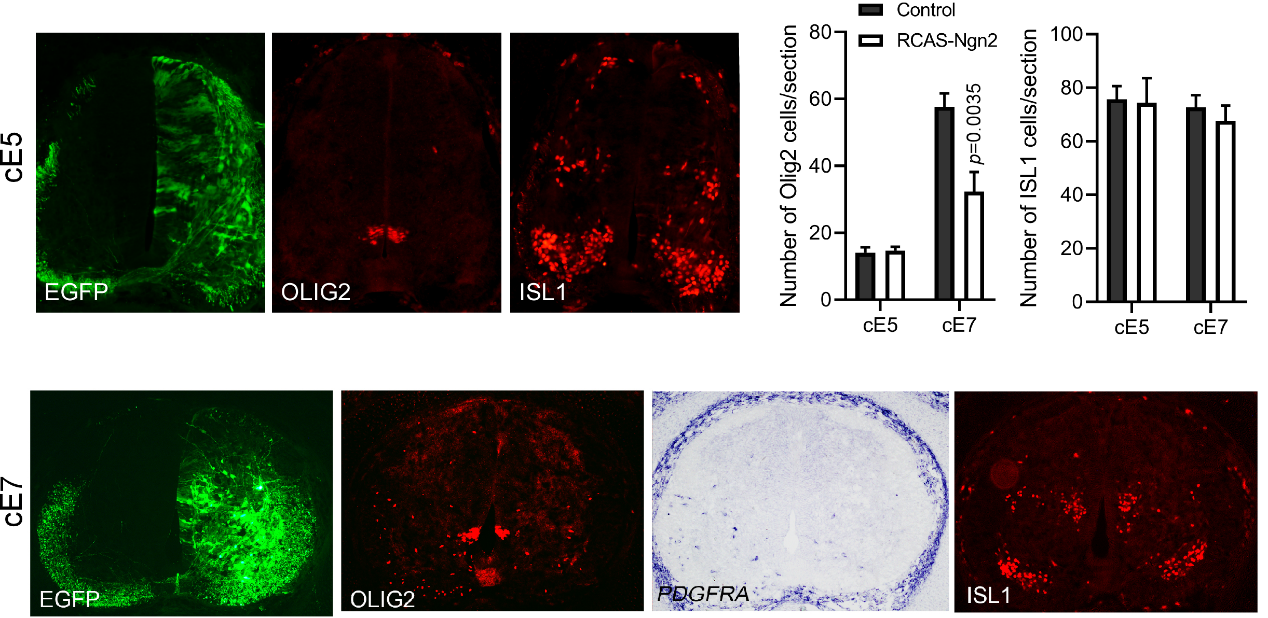


**Figure S3.** Overexpression of Ngn2 reduced the expression of Olig2 and Pdgfra at cE7. RCAS-Ngn2 was electroporated into chicken neural tube at cE2, and checked at cE5 and cE7. **: *p*<0.01.
